# A CT Reconstruction Algorithm Based on Non-Aliasing Contourlet Transform and Compressive Sensing

**DOI:** 10.1155/2014/753615

**Published:** 2014-06-30

**Authors:** Lu-zhen Deng, Peng Feng, Mian-yi Chen, Peng He, Quang-sang Vo, Biao Wei

**Affiliations:** The Key Lab of Optoelectronic Technology and Systems of the Education Ministry of China, Chongqing University, Chongqing 400044, China

## Abstract

Compressive sensing (CS) theory has great potential for reconstructing CT images from sparse-views projection data. Currently, total variation (TV-) based CT reconstruction method is a hot research point in medical CT field, which uses the gradient operator as the sparse representation approach during the iteration process. However, the images reconstructed by this method often suffer the smoothing problem; to improve the quality of reconstructed images, this paper proposed a hybrid reconstruction method combining TV and non-aliasing Contourlet transform (NACT) and using the Split-Bregman method to solve the optimization problem. Finally, the simulation results show that the proposed algorithm can reconstruct high-quality CT images from few-views projection using less iteration numbers, which is more effective in suppressing noise and artefacts than algebraic reconstruction technique (ART) and TV-based reconstruction method.

## 1. Introduction

Since computed tomography (CT) [1] technique was born in 1973, CT has been widely applied in medical diagnose, industrial nondestructive detection, and so forth. In medical CT field, how to reconstruct high-quality CT images from few-views or sparse-views data is a significant research problem. Recently, compressive sensing (CS) [2] theory has been applied in CT images reconstruction which makes it possible to reconstruct high-quality images from few-views data. In CS theory, CT images can be sparsely represented in an appropriate domain, such as gradient transform and Wavelet transform, and the quality of CT reconstructed images will be improved by some appropriate sparse representations in CT images reconstruction.

Contourlet transform [3] is proposed by Do and Vetterli in 2002, which is a sparse representation for 2D images with some properties such as multiresolution, multiscale, and multidirection. Contourlet transform can also get important smooth contour features of the image with few data, but there is frequency aliasing in Contourlet transform. Sharp frequency localization Contourlet transform [4] is firstly proposed by Lu and Do in 2006 and Feng et al. introduced a detailed explanation and construction in 2009 which is named as non-aliasing Contourlet transform (NACT) [5]. NACT which can eliminate the frequency aliasing in Contourlet transform is more efficient in capturing geometrical structure and can represent image sparser than traditional Contourlet transform.

To solve the optimization problem in CT images reconstruction based on CS, Goldstein and Osher proposed Split-Bregman [6] method, which is derived from Bregman [7] iteration and can accelerate iteration convergence and produce better reconstruction results. Split-Bregman method uses an intermediate variable to split *L*_1_ regularization and *L*_2_ regularization into two equations, where *L*_2_ and *L*_1_ regularization equation can be solved by steepest descent method and thresholding algorithm, respectively. Based on Split-Bregman method, Vandeghinste et al. proposed Split-Bregman-based sparse-view CT reconstruction approach [8]. Furthermore, an iterative CT reconstruction is proposed using shearlet-based regularization [9]. Chu et al. proposed multienergy CT reconstruction based on low rank and sparsity with the Split-Bregman method (MLRSS) [10]. Chang et al. proposed a few-view reweighted sparsity hunting (FRESH) method for CT images reconstruction [11].

In this paper, we propose a CT reconstruction algorithm based on NACT and compressive sensing which tries to explore the sparse capability of NACT in order to reconstruct high-quality CT images. In the following section, the proposed algorithm will be introduced. In the third section, we will analyze the experimental results and discuss relevant issues. In the last section, Section 4, we will conclude the paper.

## 2. Theory and Method

### 2.1. CT Reconstruction Theory Based on Compressive Sensing

Theoretically, the mathematical CT model can be expressed as:

(1) $$\begin{array}{c} Af=p, \end{array}$$

where *A* is the system matrix, *f* is the original image, and *p* is the projection data. Traditional CT reconstruction algorithms such as filtered backprojection (FBP) [12] and algebraic reconstruction technique (ART) [13] cannot reconstruct high quality CT images with the sparse sampling or limited projection data.

In 2006, Candes and Donoho put forward the CS theory which makes it possible to get high quality CT images with sparse projection data. The main idea of CS is that a signal can be reconstructed with far less sampled frequency than required by conventional Nyquist-Shannon sampling frequency, if the image has a sparse/compressible representation in a transform domain.

Compressive sensing theory can be expressed by the following equation:

(2) min⁡  ||y||0 s.t.  p=Af=AΦHy,

where Φ is a orthogonal transform, Φ^*H*^ is the corresponding inverse transform, and *f* is the CT image to be reconstructed and has a special relationship with Φ^*H*^; that is, *f* = Φ^*H*^ *y*. *p* is the projection data of *f* through matrix *A*.

Inspired by CS theory, Sidky et al. proposed a total variation (TV-) based CT reconstruction algorithm using gradient operator as the sparse representation [14], in which TV is defined as follows:

(3) ||f||TV=∑|∇f|=∑s,t(∇xf)2+(∇yf)2=∑s,t(fs,t−fs−1,t)2+(fs,t−fs,t−1)2,

where ∇*f* represents gradient operator of an image *f*.

### 2.2. Non-Aliasing Contourlet Transform

The traditional sparse representation, such as gradient operator and Wavelet transform [15], cannot get ideal sparse representation of CT images. In 2002, Ni et al. proposed Contourlet transform [16] which can utilize intrinsic structure information of image to represent images more efficiently compared with Wavelet transform. However, suffering from frequency aliasing, Contourlet transform does not show good performance in image denoising, fusion, and enhancement. In order to solve this problem, a new multiscale analysis method, named non-aliasing Contourlet transform (NACT) was proposed. NACT consists of non-aliasing pyramidal filter banks (NPFB) and directional filter banks (DFB). NPFB contains two different filter banks: *L*_0_(*ω*), *D*_0_(*ω*) and *L*_1_(*ω*), *D*_1_(*ω*). *L*_0_(*ω*) and *L*_1_(*ω*) mean low-pass filters. *D*_0_(*ω*) and *D*_1_(*ω*) mean high-pass filters. The relationships of two different filter banks are as follows:

(4) $$\begin{array}{c} D_{1}^{2}\left(\omega \right)+\frac{L_{1}^{2}\left(\omega \right)}{4}=1,\\ D_{0}^{2}\left(\omega \right)+L_{0}^{2}\left(\omega \right)=1. \end{array}$$

We assume that *ω*_*p*,0_ and *ω*_*s*,0_ represent pass-band frequency and stop-band frequency of *L*_0_(*ω*), respectively. Accordingly, *ω*_*p*,1_ and *ω*_*s*,1_ represent pass-band frequency and stop-band frequency of *L*_1_(*ω*), respectively. In order to eliminate frequency aliasing, the filter parameters should meet (1)  *ω*_*s*,1_ < *π*/2; (2)  (*ω*_*p*,0_ + *ω*_*s*,0_)/2 = *π*/2 and (*ω*_*p*,1_ + *ω*_*s*,1_)/2 = *π*/4; (3)  *ω*_*s*,0_ ≤ *π* − *a* and *ω*_*s*,1_ ≤ (*π* − *a*)/2, where *a* is the maximum width of mixing ingredients in DFB [3].

**Figure 1:**
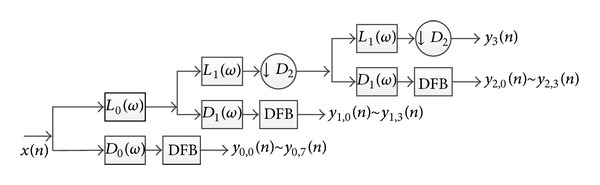
Flowchart of 3 levels of decomposition of NACT.

As a sparse representation approach, NACT integrate NPFB and DFB which can decompose image into multidirection and multiresolution. NPFB decomposes image into an approximation subband and several detail subbands with different resolutions; DFB decomposes the detail subbands into directional subbands. The process of decomposition with 3 levels is shown in Figure 1. We will use “9-7” filter and “pkva” directional filter bank [17] in the study.

### 2.3. Split Bregman Method

In CS theory, *L*_0_ norm is the most ideal regularization norm, but it is difficult to solve equations and easily interfered by noise in CT reconstruction, so *L*_0_ norm is commonly replaced by *L*_1_ norm. Then the reconstruction problem depicted by (2) can be converted into

(5a) $$\begin{array}{c} \min\;{||y||}_{0}\;\text{s}.\text{t}.\;p=Af=A\Phi^{H}y, \end{array}$$

(5b) $$\begin{array}{c} \min\;{||\Phi f||}_{1}\;\text{s}.\text{t}.\;p=Af=A\Phi^{H}y, \end{array}$$

where *y* = Φ*f*, Φ is the sparse transform which is normally used as Wavelet transform, Curvelet transform, gradient operator, and so forth.

Furthermore, (5b) can be converted into

(6) $$\begin{array}{c} f=\underset{f}{\arg\;\min}\;{||\Phi f||}_{1}+\lambda {||Af-p||}_{2}^{2}, \end{array}$$

where *λ* is penalty function weight.

In order to solve (6), Goldstein and Osher proposed Split-Bregman method [6], using an intermediate variable to split *L*_1_ regularization and *L*_2_ regularization into two equations; *L*_2_ regularization equation can be solved by gradient descent method and *L*_1_ regularization equation can be solved by thresholding algorithm. Split-Bregman method contains the following three iteration steps:

**Step 1 .**

(7) $$\begin{array}{c} f^{k+1}=\underset{f}{\arg\;\min}\;\lambda {||Af-p||}_{2}^{2}+\mu {||d^{k}-\Phi f-b^{k}||}_{2}^{2}. \end{array}$$

**Step 2 .**

(8) $$\begin{array}{c} d^{k+1}=\underset{d}{\min}\;{||d||}_{1}+\mu {||d^{k}-\Phi f^{k+1}-b^{k}||}_{2}^{2}. \end{array}$$

**Step 3 .**

(9) $$\begin{array}{c} b^{k+1}=b^{k}+\left(\Phi f^{k+1}-d^{k+1}\right), \end{array}$$

where *k* is the Split-Bregman iteration index, *μ* is convergence parameter, and *d* and *b* are intermediate variables, with which each subproblem can be solved easily.

### 2.4. Proposed Algorithm

According to aforementioned methods, we propose a CT reconstruction algorithm based on NACT and compressive sensing method which can be defined as a constrained form (10) or an unconstrained form (11) as follows:

(10) min⁡ ||f||TV+||Φf||1.s.t. ||Af−p||22<σ2

(11) $$\begin{array}{c} \;\min\;{||f||}_{\text{TV}}+{||\Phi f||}_{1}+\lambda {||Af-p||}_{2}^{2}. \end{array}$$

Applying the Split-Bregman method to (11), we have the following three iteration steps:

**Step 1 .**

(12) fk+1=arg min⁡f⁡ λ||Af−p||22+γ||dk−∇f−bk||22 +μ||dφk−Φf−bφk||22.

**Step 2 .**

(13) dk+1=min⁡d⁡ ||d||1+γ||dk−∇fk+1−bk||22dφk+1=min⁡dφ⁡ ||dφ||1+μ||dφk−Φfk+1−bφk||22.

**Step 3 .**

(14) bk+1=bk+(∇fk+1−dk+1)bφk+1=bφk+(Φfk+1−dφk+1),

where *γ* is convergence parameter and *d*_*φ*_ and *b*_*φ*_ are intermediate variables.

The steepest descent method is applied to solve (12). The derivative of (12) is calculated as follows:

(15) g[n,m+1] =2λAmT(Amfm−pm)−2γ∇T(dk−∇fm−bk)  −2μΦT(dφk−Φfm−bφk)fm+1=fm+αg[n,m+1],

where *n* denotes the iteration index of the steepest descent method, *m* = 2,…, *N*_data_ denotes the projection angles, *A*_*m*_ is* m*th row vector, and system matrix *A* includes *N*_data_ row vector *A*_*m*_. Accordingly, *N*_data_ row vectors *p*_*m*_ compose the projection-data vector *p*, *α* is an appropriate step size. The ART method is used to get initial image of iteration. Equation (13) can be explicitly computed as (16) using the shrinkage operator as follows:

(16) $$\begin{array}{c} d^{k+1}=\text{shrink}\left(\nabla f^{k+1}+b^{k},\frac{1}{\lambda }\right)\\ d_{\varphi }^{k+1}=\text{shrink}\left(\Phi f^{k+1}+b_{\varphi }^{k},\frac{1}{\mu }\right). \end{array}$$

We now describe the iterative steps of the proposed algorithm. The iteration process contains two loops, the outside loop operate ART and the inside loop solve the optimization problem which is constrained by TV and NACT. The outside loop is labeled by *n* and the inside loop is labeled by *k*. The steps comprising each loop are the DATA-step, which enforces consistency with the projection data; the POS-step, which ensures a nonnegative image. We use *f* ^(ART-DATA)^[*n*, *m*] to denote the *m*th DATA-step subiteration with the *n*th iteration and *f* ^(ART-POS)^[*n*] to denote the POS-step with the *n*th iteration in the outside loop. We use *f* ^(NACTTV-DATA)^[*k*, *m*] to denote the *m*th DATA-step subiteration with the *k*th iteration and *f* ^(NACTTV-POS)^[*k*] to denote the POS-step with the *k*th iteration in the inside loop. The steps of the algorithm are as follows:

(A) initialization:
  (17) $$\begin{array}{c} n=1,\;f^{\left(\text{ART-DATA}\right)}\left[n,1\right]=0; \end{array}$$
(B) data projection iteration, for *m* = 2,…, *N*_data_:
  (18) f(ART-DATA)[n,m]=f(ART-DATA)[n,m−1] +Ampm−Am·f(ART-DATA)[n,m−1]Am·Am;
(C) positivity constraint:
  (19) (fi,j)(ART-POS)[n]={(fi,j)(ART-DATA)[n,Ndata](fi,j)(ART-DATA)[n,Ndata]≥0,0(fi,j)(ART-DATA)[n,Ndata]<0;
(D) initialization of Split-Bregman:
  (20) $$\begin{array}{c} k=1,\\ d\left(n\right)={||f^{\left(\text{ART-DATA}\right)}[n,1]-f^{\left(\text{ART-POS}\right)}[n]||}_{2},\\ f^{\left(\text{NACTTV-DATA}\right)}\left[k,1\right]=f^{\left(\text{ART-POS}\right)}\left[n\right]\\ d_{x}^{k}=\nabla_{x}f^{\left(\text{ART-POS}\right)}\left[n\right],\\ d_{y}^{k}=\nabla_{y}f^{\left(\text{ART-POS}\right)}\left[n\right],\\ d_{\varphi }^{k}=\Phi f^{\left(\text{ART-POS}\right)}\left[n\right],\\ b_{x}^{k}=b_{y}^{k}=b_{\varphi }^{k}=0; \end{array}$$
(E) iteration for *m* = 2,…, *N*_data_:
  (21) dp=Amf(NACTTV-DATA)[k,m−1]−pm−1g[k,m−1] =2λAmdp−2γ∇xT(dxk−∇xf−bxk)  −2γ∇yT(dyk−∇yf−byk)  −2μΦT(dφk−Φf−bφk)|f=f(NACTTV-DATA)[k,m−1],g^[k,m−1]=g[k,m−1]|g[k,m−1]|,f(NACTTV-DATA)[k,m] =f(NACTTV-DATA)[k,m−1]−ad(n)g^[k,m−1];
(F) positivity constraint:
  (22) (fi,j)(NACTTV-POS)[k+1]={(fi,j)(NACTTV-DATA)[k,Ndata](fi,j)(NACTTV-DATA)[k,Ndata]≥0,0(fi,j)(NACTTV-DATA)[k,Ndata]<0;
(G) update *d*_*x*_, *d*_*y*_, *d*_*φ*_, *b*_*x*_, *b*_*y*_, *b*_*φ*_, increase *k*, and return to step (E) until *k* = *K*_NACTTV_ as follows:
  (23) dxk+1=shrink(∇xf(NACTTV-POS)[k+1,1]+bxk,1λ)byk+1=shrink(∇yf(NACTTV-POS)[k+1,1]+byk,1λ)dφk+1=shrink(Φf(NACTTV-POS)[k+1,1]+bφk,1μ)bxk+1=bxk+(∇xf(NACTTV-POS)[k+1,1]−dxk+1)byk+1=byk+(∇yf(NACTTV-POS)[k+1,1]−dyk+1)bφk+1=bφk+(Φf(NACTTV-POS)[k+1,1]−dφk+1);
(H) initialize next loop:
  (24) $$\begin{array}{c} f^{\left(\text{ART-DATA}\right)}\left[n+1,1\right]=f^{\left(\text{NACTTV-POS}\right)}\left[K_{\text{NACTTV}},1\right]; \end{array}$$

increase *n* and return to step (B). The iteration is stopped when ||*Af*−*p*||_2_^2^ < *σ* ^2^. In our study, we selected *λ* = 1000, *γ* = 30, *μ* = 30, *a* = 0.2 and *K*_NACTTV_ = 10, which can strike a good balance in the steepest descent and generate good reconstruction results in the experiments.

## 3. Experimental Results

### 3.1. The Image Quality Evaluation

This paper uses the root mean square errors (RMSE) and universal quality index (UQI) [18] to evaluate the quality of the reconstructed images.

RMSE is the most widely applied way to evaluate image quality, and RMSE is defined as

(25) $$\begin{array}{c} \text{RMSE}=\sqrt{\frac{1}{M\times N}\underset{0\leq i<N}{\sum }\;\underset{0\leq j<M}{\sum }{\left(f_{i,j}-f_{i,j}^{R}\right)}^{2}}, \end{array}$$

where *f*_*i*,*j*_ is the pixel value of original image and *f*_*i*,*j*_^*R*^ is the pixel value of reconstructed image.

Wang and Bovic proposed UQI mode which evaluates images distortion problem including correlation distortion, brightness distortion, and contrast distortion. The value of UQI is between −1 and 1. When the reconstructed image is the same as the original image, the value of UQI is 1. UQI is defined as

(26) $$\begin{array}{c} \text{UQI}=\frac{4\sigma_{\overset{-}{f},{\overset{-}{f}}^{R}}\overset{-}{f}\times {\overset{-}{f}}^{R}}{\left(\sigma_{f}^{2}+\sigma_{f^{R}}^{2}\right)\left[{\left(\overset{-}{f}\right)}^{2}+{\left({\overset{-}{f}}^{R}\right)}^{2}\right]}, \end{array}$$

where

(27) $$\begin{array}{c} \overset{-}{f}=\frac{1}{M\times N}\underset{0\leq i<N}{\sum }\;\underset{0\leq j<M}{\sum }f_{i,j},\\ {\overset{-}{f}}^{R}=\frac{1}{M\times N}\underset{0\leq i<N}{\sum }\;\underset{0\leq j<M}{\sum }f_{i,j}^{R},\\ \sigma_{f}^{2}=\frac{1}{M\times N-1}{\underset{0\leq i<N}{\sum }\;\underset{0\leq j<M}{\sum }\left(f_{i,j}-\overset{-}{f}\right)}^{2},\\ \sigma_{f^{R}}^{2}=\frac{1}{M\times N-1}{\underset{0\leq i<N}{\sum }\;\underset{0\leq j<M}{\sum }\left(f_{i,j}^{R}-{\overset{-}{f}}^{R}\right)}^{2},\\ \sigma_{f,f^{R}}=\frac{1}{M\times N-1}\underset{0\leq i<N}{\sum }\;\underset{0\leq j<M}{\sum }\left(f_{i,j}-\overset{-}{f}\right)\left(f_{i,j}^{R}-{\overset{-}{f}}^{R}\right). \end{array}$$

### 3.2. Numerical Simulation

**Figure 2:**
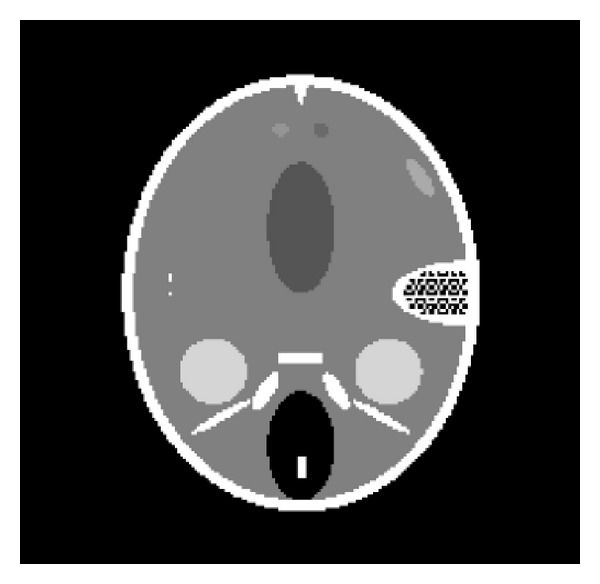
Head phantom.

In this section, a head phantom as shown in Figure 2 is used to reconstruct and compare by 3 different methods: ART, ART-TV, and our proposed algorithm (SpBr-NACT). The size of phantom image is 200 × 200. We assume that the CT system was viewed as in a typical pencil-beam geometry, and the scanning range was from 1° to 360° with a *θ* angular increment; projection angles can be indicated as

(28) $$\begin{array}{c} \theta_{i}=1+360\times \frac{\left(i-1\right)}{N_{\text{view}}},\;i=1,2,\ldots ,\frac{N_{\text{view}}}{2}\\ \theta_{i}=182+360\times \frac{\left(i-N_{\text{view}}/2\right)}{N_{\text{view}}},\\ i=\frac{N_{\text{view}}}{2},\frac{N_{\text{view}}}{2}+1,\ldots ,N_{\text{view}}. \end{array}$$

**Figure 3:**
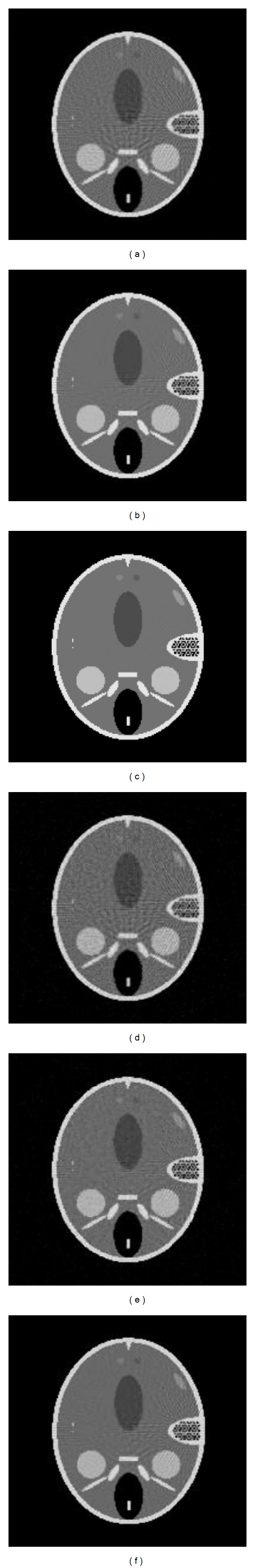
The reconstructed images using three different reconstruction algorithms from the noise-free and noisy data. Top row is for noise-free data and bottom row is for noisy data. (a) and (d) are reconstructed by ART, (b) and (e) are reconstructed by ART-TV, and (c) and (f) are reconstructed by SpBr-NACT method.

**Figure 4:**
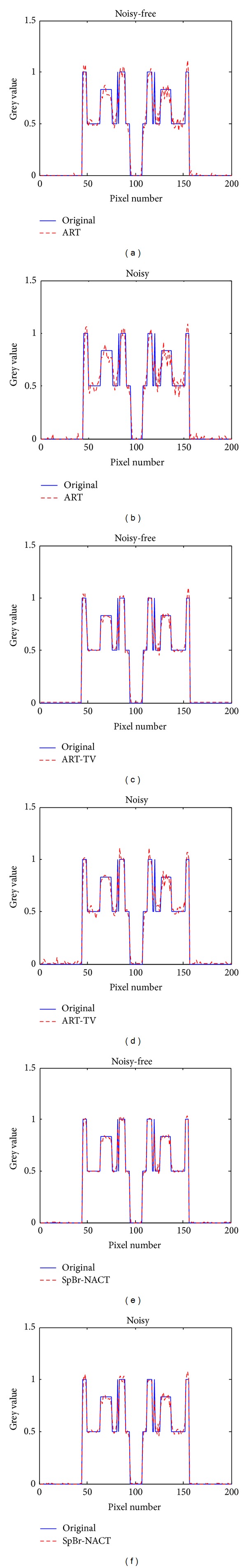
The profile of line 140 in different reconstructed images. Left column is for noise-free date and right column is for noisy data. (a) and (b) are for ART method; (c) and (d) are for ART-TV method; (e) and (f) are for SpBr-NACT method.

In the simulation, we reconstruct the head phantom from noise-free and noisy projection data. To obtain noisy projection data, we add 10 dB Gaussian noise into noise-free projection data. Projection number *N*_view_ is 60, and iteration numbers for all reconstruction algorithms are 50. The reconstructed images are shown in Figure 3 and the profile of line 140 in different reconstructed images is plotted in Figure 4.

From Figures 3 and 4, we can see that the reconstructed images using ART and ART-TV methods contain a lot of noise and artifacts, while the reconstructed images using SpBr-NACT method contain less noise and artifacts and have clearer edges.

Table 1 lists all the RMSE and UQI calculated from reconstructed images. It is obvious that the RMSE of reconstructed images using SpBr-NACT method is much smaller than that of reconstructed images using ART and ART-TV methods; the UQI is much bigger. Thus SpBr-NACT method can reconstruct higher quality images.

**Figure 5:**
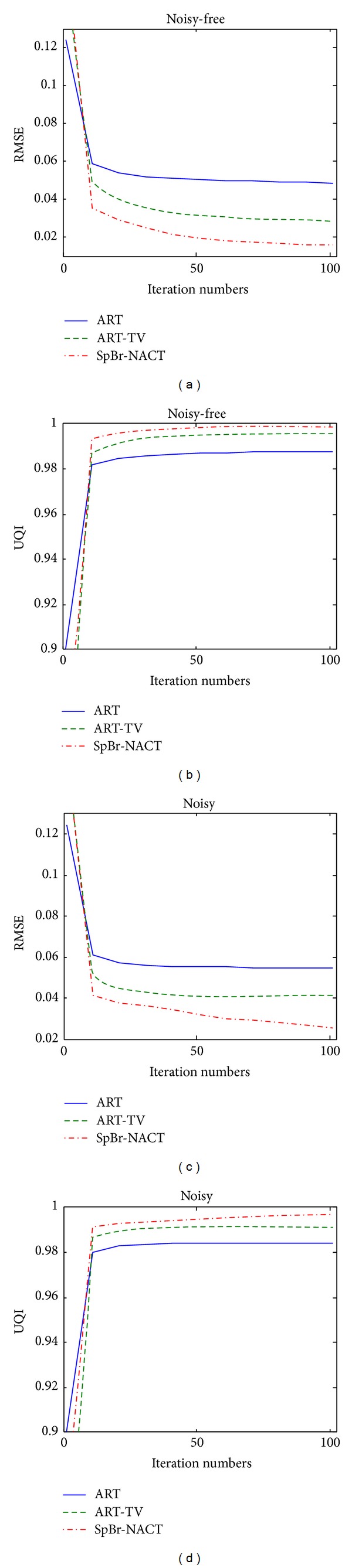
The relationship of RMSE and UQI with respect to iteration number. (a) and (b) are the RMSE and UQI of reconstructed images from noise-free data with ART, ART-TV, and SpBr-NACT method, respectively. (c) and (d) are the RMSE and UQI of reconstructed images from noisy data with ART, ART-TV, and SpBr-NACT method, respectively.

**Figure 6:**
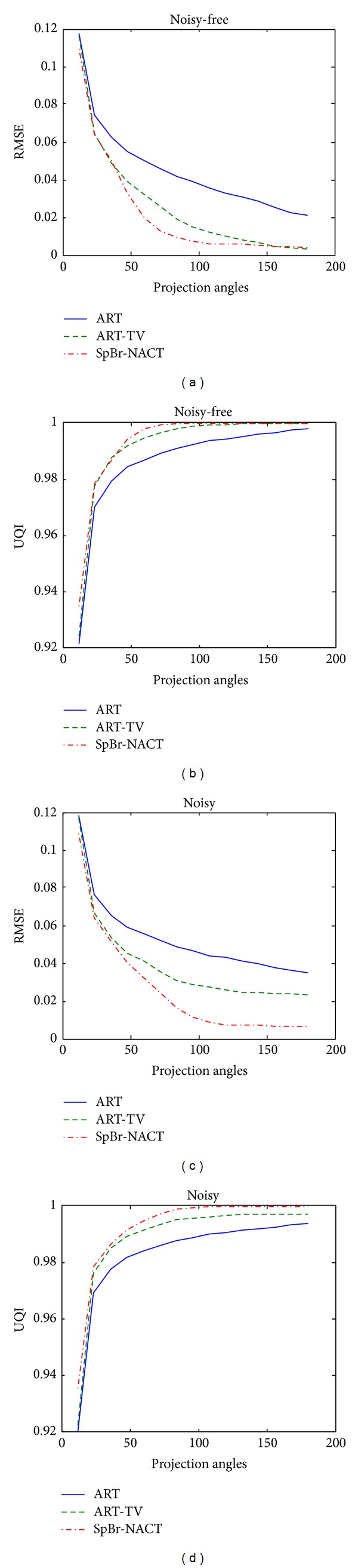
The relationship of RMSE and UQI with respect to projection number *N*_view_. (a) and (b) are the RMSE and UQI of reconstructed images from noise-free data with ART, ART-TV, and SpBr-NACT method, respectively. (c) and (d) are the RMSE and UQI of reconstructed images from noisy data with ART, ART-TV, and SpBr-NACT method, respectively.

Figure 5 plots the change of RMSE and UQI with respect to iteration number.  Figure 6 plots the change of RMSE and UQI with respect to projection number *N*_view_. In both figures, ART, ART-TV, and the proposed SpBr-NACT approach are used to reconstruct images from noise-free and noisy data. The blue-solid line is for ART, the green-dashed line is for ART-TV, and the red dashed line is SpBr-NACT. For Figure 5, the projection number is fixed and *N*_view_ is 60. For figure 6, the iteration number is fixed and equals 50. From both Figures, it is easy to find that with the increase of projection number or iteration number, SpBr-NACT approach can always get the minimum RMSE and maximum UQI which means that the quality of reconstructed images with SpBr-NACT is better than those with ART and ART-TV. And also we see from Figure 5 when the iteration number is relatively small, 3 methods that almost have the same RMSE and UQI, which implies that our proposed method has no advantage if the iteration step does not converge.

## 4. Conclusion

In this study, we proposed a CT reconstruction algorithm based on NACT and compressive sensing. The experimental results demonstrate that the proposed method can reconstruct high-quality images from few-views data and has a potential for reducing the radiation dose in clinical application. In the further research, we will try to explore more directional information from NACT so as to improve the performance of SpBr-NACT algorithm, especially when the projection number is far more below what we setup in the current experiment.

## Figures and Tables

**Table 1:** RMSE and UQI of reconstructed images using three different algorithms.

	RMSE			UQI		
Methods	ART	ART-TV	SpBr-NACT	ART	ART-TV	SpBr-NACT
Noisy-free	0.0502	0.0321	0.0196	0.9869	0.9947	0.9980
Noisy	0.0554	0.0406	0.0318	0.9839	0.9914	0.9948
